# FUCCI-Based Live Imaging Platform Reveals Cell Cycle Dynamics and Identifies Pro-proliferative Compounds in Human iPSC-Derived Cardiomyocytes

**DOI:** 10.3389/fcvm.2022.840147

**Published:** 2022-04-25

**Authors:** Francesca Murganti, Wouter Derks, Marion Baniol, Irina Simonova, Palina Trus, Katrin Neumann, Shahryar Khattak, Kaomei Guan, Olaf Bergmann

**Affiliations:** ^1^Center for Regenerative Therapies Dresden, TU Dresden, Dresden, Germany; ^2^Karolinska Institute, Cell and Molecular Biology (CMB), Stockholm, Sweden; ^3^Royal College of Surgeons Ireland (RCSI) in Bahrain, Adliya, Bahrain; ^4^Institute of Pharmacology and Toxicology, TU Dresden, Dresden, Germany

**Keywords:** cardiomyocyte, cell cycle activity, cell cycle indicator, fluorescence ubiquitination cell cycle indicator, induced pluripotent stem cell, clonidine, polyploidy

## Abstract

One of the major goals in cardiac regeneration research is to replace lost ventricular tissue with new cardiomyocytes. However, cardiomyocyte proliferation drops to low levels in neonatal hearts and is no longer efficient in compensating for the loss of functional myocardium in heart disease. We generated a human induced pluripotent stem cell (iPSC)-derived cardiomyocyte-specific cell cycle indicator system (TNNT2-FUCCI) to characterize regular and aberrant cardiomyocyte cycle dynamics. We visualized cell cycle progression in TNNT2-FUCCI and found G2 cycle arrest in endoreplicating cardiomyocytes. Moreover, we devised a live-cell compound screening platform to identify pro-proliferative drug candidates. We found that the alpha-adrenergic receptor agonist clonidine induced cardiomyocyte proliferation *in vitro* and increased cardiomyocyte cell cycle entry in neonatal mice. In conclusion, the TNNT2-FUCCI system is a versatile tool to characterize cardiomyocyte cell cycle dynamics and identify pro-proliferative candidates with regenerative potential in the mammalian heart.

## Introduction

Heart regeneration in mammals is restricted to the neonatal period when cardiomyocytes can still proliferate (1–3). Cardiomyocyte proliferation gradually declines after birth and remains at a low level in adulthood (4, 5). Although multiple factors, including changes in metabolism (6), extracellular matrix (7), and endocrine signaling (8), contribute to this loss of proliferative activity, the exact underlying mechanism that drives cardiomyocyte cell cycle exit still needs further elucidation.

While cardiomyocyte proliferation declines, aberrant cell cycle activity becomes more dominant, leading to multinucleation and endoreplication during the first postnatal weeks (1). The increase in polyploidy is implicated in the loss of regenerative capacity in postnatal hearts (9). To date, it is not clear which cues determine the fate of cycling cells and how one can direct non-productive cell cycle activity to cytokinesis and proliferation. Moreover, this non-productive cell cycling has made the identification of cardiomyocyte proliferation challenging and has previously led to misinterpretation of the cardiomyocyte renewal capacity (10).

The Fluorescence Ubiquitin Cell Cycle Indicator (FUCCI) system is a genetically encoded, protein-based two-color cell cycle indicator that allows for the identification of cycling and non-cycling cells and relies on the ubiquitination and degradation of the cell cycle regulators Cdt1 and Geminin (11, 12). The combination of FUCCI cell cycle indicators with time-lapse microscopy can unequivocally determine whether the outcome of the cardiomyocyte cell cycle is productive (cytokinesis) or non-productive (endoreplication) (13–16).

Human induced pluripotent stem cell-derived cardiomyocytes (hiPSC-derived CMs) can currently be robustly generated using established protocols (17), diminishing the need for cardiomyocytes isolated from rodents, which do not fully recapitulate human physiology.

Depending on their level of maturity, hiPSC-derived CMs still have the capacity to proliferate (18). Thus, hiPSC-derived CMs represent an ideal model system to study the kinetics and regulation of human cardiomyocyte proliferation.

Here, we generated a troponin T2-FUCCI (TNNT2-FUCCI) hiPSC line and showed aberrant cell cycle kinetics with G2 arrest in endoreplicating cardiomyocytes compared to those undergoing proliferation and multinucleation. Moreover, we investigated our TNNT2-FUCCI hiPSC-derived CMs with an autophagy compound library in a live-cell screening approach and identified the alpha-adrenergic agonist clonidine to promote cardiomyocyte proliferation in hiPSC-derived CMs and cell cycle entry in mouse neonatal cardiomyocytes (mNCMs).

## Methods

### Generation of TNNT2-FUCCI hiPSC

The pCAG-Fucci2a plasmid (the RIKEN Center for Life Science Technologies, RDB13080) was obtained through RIKEN (19, 20). mCherry was kindly provided by Shaner et al. (21). The Fucci2a portion (mCherry-hCdt1-T2A-mVenus-hGem) was PCR amplified and cloned in frame downstream of the TNNT2 start codon into a custom synthesized (Thermo Fisher Scientific) plasmid backbone containing the ColE1 origin of replication, ampicillin resistance, 1,000 bp 5′ and 980 bp 3′ homology arms to the hTNNT2 (NM_000364.4) transcription start site, IRES-Puro and an FRT-flanked PGK-hygromycin selection cassette to generate the targeting vector using the NEBuilder High-Fidelity DNA Assembly Cloning Kit (NEB, E5520). Twenty-one bp downstream of the start codon, including the sgRNA target sites, was excluded from the targeting vector to protect it from CRISPR/Cas9-induced double-strand breaks. The resulting targeting vector was verified by Sanger sequencing, linearized by restriction with SspI and SfiI enzymes and phenol–chloroform-extracted. Two different sgRNAs (*spacer sequences below*), each targeting the region behind the start codon of hTNNT2 that was excluded from the targeting vector, were transcribed *in vitro* using an EnGen® sgRNA Synthesis Kit (NEB, E3322S). The generation and characterization of CRTD5 hiPSCs (hPSCreg, CRTDi005-B) was previously described (22). CRTD5 hiPSCs (800,000) were electroporated with 10 μg linearized targeting vector, 1 μg of each sgRNA and 60 pmol Cas9-NLS protein (EnGen® Spy Cas9 NLS, NEB, M0646 M) with the Lonza 4D X-unit, pulse CB-150 and Primary Cell 4D-Nucleofector Kit L (V4XP-3024, Lonza). Transfected cells were seeded at clonal density in dishes coated with hESC-qualified Matrigel (Corning Life Sciences, 354277) in mTeSR1 medium (StemCell Technologies, 85850) supplemented for 3 days with 10 μM Rock inhibitor Y-27632 (StemCell Technologies, 78003). Cells were selected with 50 μg/ml hygromycin B (Thermo Fisher Scientific, 10687010) starting on Day 3 after nucleofection for 7 days, and resistant colonies were manually selected, clonally expanded and screened for correctly targeted clones by colony PCRs amplifying the 5′ and 3′ junction of the targeted alleles from outside of the homology arms into the insert as well as the presence of an intact second allele. Heterozygously targeted clones were selected, and the complete insert was analyzed by Sanger sequencing. The selected complete clone CRTD5-TNNT2-FUCCI #19 was karyotyped by Giemsa banding and showed an intact chromosome set 46, XY[cp20], similar to the parental line (Supplementary Figure 1A).

hTNNT2-sgRNA#3: 5′- GACCATGTCTGACATAGAAG-A3′

hTNNT2-sgRNA#4: 5′- GGTGGTGGAAGAGTACGAGG-3′

### hiPSC Culture and Maintenance

The hiPSC line (CRTD5) generated from human fibroblasts was used in this study as a control and was obtained from the Stem Cell Engineering facility of the Center for Molecular and Cellular Bioengineering (CMCB), TU Dresden. Research with CRTD5 hiPSC was approved by the *Ethikkommission an der Technischen Universität Dresden* (BO-EK-38012020). Cells were propagated using ReLeSR (StemCell Technologies, 05873) and maintained in mTESR1^TM^ (StemCell Technologies, 85850) on Matrigel^TM^-coated plates (Corning Life Sciences, 354234) under standard culturing conditions (37 °C, 5% CO_2_). Cell cultures were routinely checked for mycoplasma using the LookOut® Mycoplasma PCR Detection Kit (Sigma Aldrich, D9307).

### hiPSC Differentiation Into Cardiomyocytes

Differentiation of CRTD5 and FUCCI-CRTD5 lines was induced by adaptation of a previously described protocol (17, 23). Briefly, undifferentiated hiPSCs were passaged into 12-well plates using Versene (Thermo Fisher Scientific, 15040066). When hiPSC culture reached 90–95% confluency, differentiation was induced using CDM3 medium (17) supplemented with the GSK3β inhibitor CHIR99021 (4 μM, Sigma, SML1046) for 48 h followed by treatment with IWP2 (5 μM, Tocris, 3533) for an additional 48 h. After, cells were cultured in CDM3 medium. At Day 15 of differentiation, hiPSC-derived CMs were gently detached from the plate by incubating with 1 mg/ml collagenase B dissolved in CDM3 medium for 30 min at 37°C. hiPSC-derived CMs were further dissociated using 0.25% trypsin-EDTA for 5 min at 37°C. The reaction was stopped by adding a double volume of stop medium (80% CDM3, 20% FBS, ROCK inhibitor). Cells were plated at a density of 1,000,000 cells/well into Matrigel-coated 12-well plates. CDM3 was exchanged every second day until further analysis.

### Imagestream-X Analysis

hiPSC-derived CMs were detached from the plate by incubating with 1 ml of TrypLE (Thermo Fisher Scientific, 12604013) for 5 min at 37°C. A double volume of PBS was added to stop the reaction, and the cells were passed through a 100 μm cell strainer. hiPSC-derived CMs were centrifuged at 200 g for 5 min, fixed with 1% PFA for 20 min and washed 3x with PBS. hiPSC-derived CMs were incubated with primary antibodies against mVenus (Biorbyt, orb334993, 1:800) and mCherry (Abcam, ab125096, 1:250) and anti-cardiac troponin T-APC (Miltenyi Biotec, 130-120-403, 1:50) in blocking buffer (PBS, 4% donkey serum, 0.1% Triton X-100 in PBS, 2 mM EDTA) for 2 h at RT. After washing, the cells were incubated with the secondary antibodies anti-goat Alexa Fluor® 488 (Jackson ImmunoResearch, 705-546-147, 1:500), anti-rabbit Alexa Fluor® 555 (Abcam, ab150062, 1:500) and Hoechst 33342 (Thermo Fisher Scientific, H21492) for 1 h at 4°C. Cells were then washed 3x with PBS and centrifuged at 200 g for 5 min. Finally, 5 million cells were resuspended in 500 μl of FACS buffer (PBS, 2% FBS, EDTA) and kept on ice until further analysis. Cells were analyzed on Amnis ImageStream-X MkII (Luminex, United States).

### hiPSC Immunohistochemistry

Cells were fixed with 4% formaldehyde solution in PBS for 10 min and stained overnight at 4°C with primary antibodies against mVenus (1:800, Biorbyt, orb334993) and mCherry (1:250, Abcam, ab125096) and anti-cardiac troponin T (1:250, Thermo Fisher Scientific, MA5-12960) in blocking buffer (PBS, 4% donkey serum, 0.1% Triton X-100 in PBS, 2 mM EDTA). Cells were then washed 3x with PBS and incubated with the following secondary antibodies in PBS: anti-goat Alexa Fluor® 488 (1:500, Jackson ImmunoResearch, 705-546-147), anti-rabbit Alexa Fluor® 555 (1:500, Abcam, ab150062), anti-mouse Alexa Fluor® 647 (1:500, Jackson ImmunoResearch, 715-606-151) and Hoechst 33342 (Thermo Fisher Scientific, H3570).

### Measurement of Cell Area and Sarcomere Spacing

CRTD5 and FUCCI-CRTD5 cardiomyocytes at Day 25 of differentiation were first costained with antibodies against TNNT2 (mouse, Thermo Fisher Scientific, MA5-12960) and α-actinin (rabbit, ThermoFisher Scientific, 701914). Imaging of single cardiomyocytes was performed using a Zeiss LSM 700. For cell area analysis, using ImageJ-Fiji software, a defined region of interest (ROI) was defined to outline the outer edges of the cell, and the cell area was measured for each cardiomyocyte. For sarcomere spacing measurements, an ROI with at least 10 sarcomeres was defined. The intensity or the ROI shows a series of peaks that correspond to the spatial frequency of the sarcomeric pattern, and the amplitude between the peaks was determined to assess the sarcomere spacing.

### Primary mNCMs Culture

Whole litters of C57BL/6JRj mice (P0) were used for isolation of mNCMs. C57BL/6JRj mice were originally obtained from Janvier labs and bred internally in CRTD animal facilities. All procedures were approved by the local ethics committee, Landesdirektion Sachsen (TVT-1/2017). P0 mNCMs were isolated using the Neonatal Heart Dissociation kit (Miltenyi Biotec, 130-098-373) following the manufacturer's instructions. Cells were seeded in plating medium (20% M199 (Thermo Fisher Scientific, 12340030), 65% DMEM (Thermo Fisher Scientific, 31966021), 5% FBS (fetal bovine serum), and 10% HS (horse serum) at a seeding density of 35,000 cells/well in a 96-well plate coated with Matrigel (Corning Life Sciences, 354234). Cells recovered for 1 day in an incubator (37°C, 5% CO_2_).

### Live Imaging and Analysis of TNNT2-FUCCI hiPSCs

HiPSC-derived CMs at different time points post cardiomyocyte induction (0, 6, and 30 days) were imaged using a Keyence BZ-X800E microscope (Keyence, Japan). Images were acquired using brightfield, YFP and Cy3 filter sets. For time-lapse imaging, FUCCI hiPSC-derived CMs at Day 30 of differentiation were seeded in CDM3 medium at a density of 150,000 cells per well in a 24-well plate (Cellvis, P24-0-N) coated with Matrigel (Corning Life Sciences, 354234). Time-lapse imaging was performed as previously described (14). Briefly, FUCCI hiPSC-derived CMs were imaged every 20 min for 72 h, and the quantification of the mCherry and mVenus fluorescent intensities was performed using Keyence image measurement and analysis software (Keyence, Japan) and ImageJ-Fiji software. A region around the cell was first defined as the background region, and the cell nucleus was segmented using the brightfield signal. The mCherry and mVenus intensities were detected in both the background region and the nucleus. The background signal was subtracted from the nuclear intensity. Single-cell intensity data were aligned based on the peak mVenus intensity and plotted over a period of 40 h using GraphPad Prism.

### Analysis of Ploidy and Binucleation in Cardiomyocytes

Culture hiPSC-derived CMs or mNCMs were fixed with 4% formaldehyde solution in PBS for 10 min and stained overnight at 4 °C with primary antibodies against cardiac troponin I (1:500, Abcam, ab56357) and Ki-67 (CellSignaling, 9449T) in blocking buffer (PBS, 4% donkey serum, 0.1% Triton X-100 in PBS, 2 mM EDTA). Cells were then washed 3x with PBS and stained with the secondary antibodies anti-goat Alexa Fluor® 488 (Jackson ImmunoResearch, 705-546-147, 1:500), anti-rabbit Alexa Fluor® 555 (1:500, Abcam, ab150062), and Vybrant DyeCycle Violet Stain (Invitrogen, V35003) in PBS. Images were acquired using a Keyence BZ X800 fluorescence microscope (Keyence, Japan) equipped with an imaging cytometer (BZ-H4XI). Image analysis was performed in the open source software CellProfiler 4.2.1 (24). Nuclei segmentation was performed using the identify primary objects module with an adaptive thresholding method to account for background variances. Cardiac troponin I and Ki-67 intensities were measured in segmented nuclei, and thresholds were determined according to their histograms. DNA staining intensities of non-cycling (Ki-67^−^) cardiomyocyte nuclei (cardiac troponin I^+^) were measured, and ploidy levels were plotted as histograms, from which ploidy thresholds were determined (Supplementary Figure 3). The number of nuclei per cardiomyocyte was determined manually analyzing a minimum of 15 field of views for each biological replicate.

### Cell Plating and Culturing and Screen Conditions

TNNT2-FUCCI hiPSC-CMs were differentiated as described above. hiPSC-CMs were used 30 days after starting the differentiation. hiPSC-CMs were plated on 96-well glass bottom plates (Cellvis, P96-1-N) coated with Matrigel (Corning Life Sciences, 354277) at a seeding density of 4000 cells per well. Cells were seeded in a volume of 50 μl RPMI20 medium (RPMI 1640 with 20% FBS). Outer wells were left unused and filled with PBS to exclude well plate edge effects. The medium was changed the next day for RPMI 1640 (Thermo Fisher Scientific, 32404014) + B27 (Thermo Fisher Scientific, 17504044) + 0.1% FBS (Thermo Fisher Scientific, 10500064). Subsequently, hiPSC-CMs were left for 3 days to reduce the baseline level of proliferation induced by the plating medium before starting the screen.

Four days after seeding the cells, the autophagy library (ENZO, BML-2837-0100) was added to wells by total medium exchange at an end concentration of 25 μM. Then, the medium was supplemented with nontoxic concentrations of Hoechst 33342 (10 ng/ml, Thermo Fisher Scientific, H3570) and EdU (5 μM, Thermo Fisher Scientific, C10340). The compound library and controls were divided over two template plates, from which the compounds in the medium were transferred into triplicate screening plates. All pipetting was performed with automated channel pipets to avoid interwell variation. Medium with compounds was not exchanged throughout the 72 h duration of the screen.

### Image Acquisition

All images were acquired using a Keyence BZ-X800E compact fluorescence microscope equipped with live imaging cytometer (BZ-H4XI) and CO_2_ control (BZ-H4XT) modules. Live images were acquired using the 10x objective and DAPI, YFP and Cy3 filter sets. Binning was set to 3x3, the gain to 6 dB and exposure times were 28 ms for DAPI, 666 ms for YFP and 167 ms for Cy3 channels. The resulting images were 640x480 pixels. Five non-overlapping sites were imaged per well. LIVE imaging was performed at Day 0 directly after adding the compound library and every 24 h until 72 h. After the last LIVE acquisition, hiPSC-CMs were fixed by incubation with 4% formaldehyde solution in PBS for 15 min. Subsequently, an EdU click-it reaction (Thermo Fisher Scientific, C10340) was performed according to the manufacturer's protocol to visualize EdU incorporation. Images of fixed cells were acquired using DAPI and Cy5 channels, binning was set to 3x3, gain to 6 dB and exposure times were 100 ms for DAPI and 10 ms for Cy3.

### Automated Image Analysis

Automated image analysis was performed in the open source software CellProfiler 4.2.1 (24). Briefly, single channel images from DAPI, YFP, and Cy3 channels were imported into the program. In the first module, the YFP images were enhanced to reduce uneven backgrounds. Subsequently, the DAPI channel was used to identify all nuclei and assign them as primary objects. In the next modules, the intensity of mVenus and mCherry from the YFP and Cy3 channel images within the nuclei was measured, and a threshold was set to categorize the nuclei as positive (+) or negative (–) for these channels. In the following modules, we assigned all nuclei to a single category as follows: mVenus- and mCherry- nuclei: BLUE; mCherry^+^ and mVenus^−^ nuclei: RED; mCherry^+^ and mVenus^+^ nuclei: YELLOW; mVenus^+^ and mCherry^−^ nuclei: GREEN. In the last module, counts were exported into spreadsheets for further data analysis.

### Data Analysis of the TNNT2-FUCCI Screen

Data analysis was performed using the open-source data analytics software KNIME (Konstanz Information Miner) version 4.3.1. The general approach for screen analysis was followed as described by Stöter et al. (25). Briefly, CellProfiler output files were imported, and the following steps were performed: quality control, filtering, grouping, normalization, and statistics. As a first step, data from individual sites were excluded from analysis if the number of nuclei was far below (>2 STDEV) mean levels (indicating problems with focus, e.g.,). In the second step, the results from imaging sites were grouped by wells, giving mean values per well from the five sites imaged. In the next steps, the data from wells were matched with locations on the plates and the compound information. The percentages per compound were normalized to the control (100%). Next, wells were grouped by treatment, and if parameters showed STDEVs larger than half the value of the parameter, they were excluded as quality controls. Data were exported into Excel, and the main parameter (% of mVenus^+^ in all FUCCI^+^) was plotted using GraphPad Prism software.

### Clonidine Treatment of Neonatal Mice

Animals were housed in the Comparative Medicine Biomedicum (Karolinska Institutet, Stockholm) animal facility on a 12-h light/dark cycle and were provided food and water *ad libitum*. All breeding and organ collection protocols were performed in accordance with the Swedish and European Union guidelines and approved by the institutional ethics committee (Stockholms Norra Djurförsöksetiska Nämnd). C57BL/6N neonatal mice were injected subcutaneously with a volume of 0.1 ml of PBS with EdU (20 mg/kg, Invitrogen, E10187) and clonidine (60 ng/pup, Sigma, C7897). Clonidine was given for an estimated weight of 1.5 g/pup throughout the experiment; therefore, a fixed dose of 60 ng/pup was used. Neonatal mice were sacrificed at P7 by decapitation, and hearts were dissected and collected in PBS, cryoprotected in 30% sucrose and flash-frozen in isopentane.

### Immunohistochemistry Staining of P7 Neonatal Mouse Hearts

Frozen hearts were sectioned into 10 μm thick sections at the cryostat. After washing with PBS sections were fixed by incubating in 2% formaldehyde solution for 10 min. Primary antibodies against rabbit PCM-1 (1:100, Santa Cruz, sc-67204), mouse SMA-Cy3 (1:1000, SigmaAldrich, C6198), and biotinylated isolectin B4 (Vector labs, B-1205, 1:500) were diluted in blocking buffer (PBS, 4% donkey serum, 0.1% Triton X-100 in PBS, 2 mM EDTA) and sections were incubated overnight at RT. Sections were then washed 3x with PBS (15 min) and stained with matching secondary antibody anti-rabbit Alexa Fluor® 488 (1:500, Jackson ImmunoResearch, 711-546-152) and Streptavidin-Alexa Fluor 647® (1:1000, ThermoFisher, S21374) in PBS. Subsequently, Click-iT™ EdU Cell Proliferation Kit for Imaging, Alexa Fluor™ 647 or 488 dyes (ThermoFisher, C10340 and C10637) was used to detect EdU. Slides were then washed and mounted using ProLong Gold Antifade Mountant with DAPI (ThermoFisher, P36931). Images were acquired using a Keyence BZ X800 fluorescence microscope (Keyence, Japan) and Zeiss LSM 750 confocal microscopy from at least three regions of the left ventricle per heart.

### Neonatal Cardiomyocyte Isolation and Immunocytochemistry

Frozen hearts were placed on ice, cut into small pieces, fixed in four percent paraformaldehyde for 1 h, and digested for 2 h at 37°C (3.6 mg/ml collagenase B, 4.8 mg/ml collagenase D, in PBS). Cells were incubated with primary antibody against α-Actinin (Thermofisher, A7811, 1:500) and Connexin-43 (SigmaAldrich, C6219, 1:1000) for 30 min to label cardiomyocyte cytoplasm and borders. After washing with PBS, cells were resuspended with matched secondary antibodies [AF488-coupled (Abcam, ab150110, 1:1000) and AF555-coupled (Jackson Immuno Research, 711-546-152, 1:500)] for 30 minutes. Cardiomyocytes were placed on a slide with a mounting medium (Invitrogen™ ProLong™ Gold Antifade Mountant with DAPI). Images were acquired using a Keyence BZ X800 fluorescence microscope (Keyence, Japan) equipped with an imaging cytometer (BZ-H4XI). Image analysis was performed in the open source software CellProfiler 4.2.1 (24). Nuclei segmentation was performed using the identify primary objects module with an adaptive thresholding method to account for background variances. α-Actinin intensities were measured in segmented nuclei, and thresholds were determined according to their histograms. DNA staining intensities of cardiomyocyte nuclei were measured, and ploidy levels were plotted as histograms, from which ploidy thresholds were determined (Supplementary Figure 4). The number of nuclei per cardiomyocyte was determined manually analyzing a minimum of 15 field of views for each biological replicate.

## Results

### Generation and Validation of TNNT2-FUCCI in hiPSC-Derived CMs

**Figure 1:**
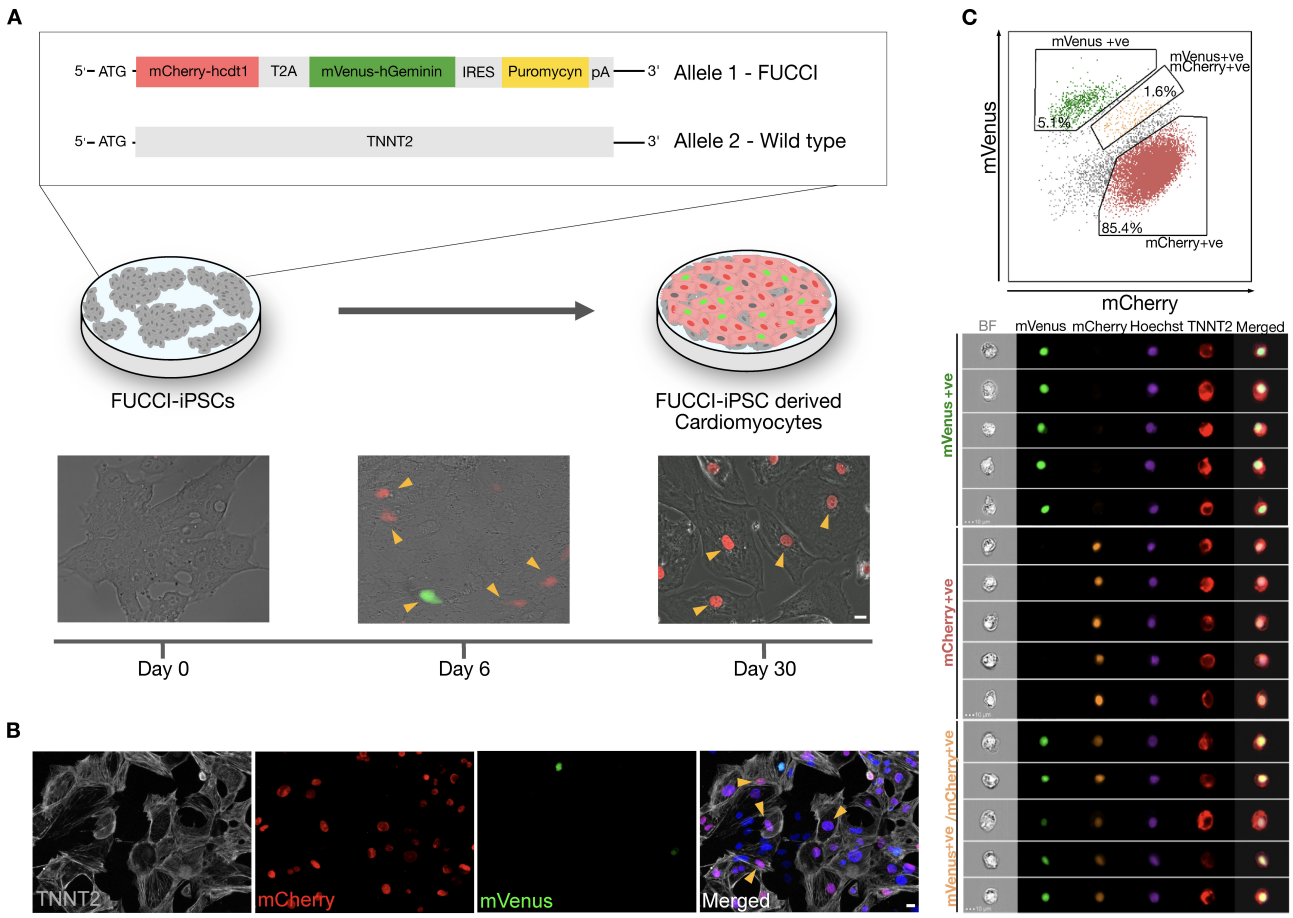
Generation and validation of TNNT2-FUCCI in hiPSC-derived CMs. **(A)** Schematic representation showing TNNT2-FUCCI reporter cell line generation and differentiation towards cardiomyocytes. Upon differentiation into cardiomyocytes, the FUCCI signal became visible at Day 6. Arrowheads indicate FUCCI^+^ nuclei. Scale bar, 20 μm. **(B)** Immunocytochemistry showing cardiomyocytes at Day 30 of differentiation. Arrowheads indicate cardiomyocytes (TNNT2^+^) that were FUCCI^+^, demonstrating the specificity of the FUCCI reporter. Scale bar, 20 μm. **(C)** Imaging flow cytometry shows TNNT2^+^ cells expressing nuclear mVenus and/or mCherry, indicating cardiomyocyte specificity of the TNNT2-FUCCI system.

We generated a TNNT2-FUCCI hiPSC line using a CRISPR–Cas9 approach (see Methods). TNNT2-FUCCI hiPSCs expressed the FUCCI construct under the control of the cardiomyocyte-specific TNNT2 promoter (Figure 1A and Supplementary Figure 1A). The generated TNNT2-FUCCI hiPSC line showed a normal karyotype, and pluripotency characterization showed high expression of bona fide pluripotency markers and could generate three germ layers (Supplementary Figures 1B–D). FUCCI fluorescence (mCherry/mVenus) became visible from Day 6 post differentiation into cardiomyocytes (Figure 1A). At Day 30, in most cells, FUCCI signal was detected (Figure 1A), and all FUCCI-expressing cells were TNNT2^+^ (Figure 1B). To further confirm the cardiomyocyte-specific expression of TNNT2-FUCCI fluorescence, we used imaging flow cytometry. We analyzed 50,000 cells defined by their TNNT2 expression and found that 85.4% were mCherry-Cdt1^+^ (G0/G1 phase), 5.1% were mVenus-geminin^+^ (G2/M phase) and 1.6% of the cardioymocytes were both mCherry-Cdt1 and mVenus-geminin^+^ (G1/S phase) (Figure 1C). Importantly, we found no FUCCI expression in the TNNT2^−^ cell fraction. However, some signal in the TNNT2^−^ fraction detected in the green and red fluorescence channels could be assigned to autofluorescence (Supplementary Figure 2A).

To demonstrate that the TNNT2-FUCCI signal reliably indicates the cell cycle status of cardiomyocytes, we costained FUCCI cardiomyocytes with cyclin-dependent kinase-1 (CDK1) and found a complete overlap of mVenus^+^ and CDK1^+^ nuclei, supporting that mVenus^+^ cardiomyocytes indeed represent cells in the S/G2/M phase of the cell cycle (Supplementary Figure 2B).

TNNT2-FUCCI cardiomyocytes showed a similar sarcomere spacing pattern (1.97 μm ± 0.06 μm SEM) and cell size (2525.4 μm^2^ ± 259.2 μm^2^ SEM) as controls (Supplementary Figures 2C,D), suggesting that the integration of the TNNT2-FUCCI knock-in did not compromise the functionality of the TNNT2-FUCCI cardiomyocytes.

In summary, we showed that TNNT2-FUCCI expression is limited to cardiomyocytes and reliably detects cell cycle progression.

### Live Cell Imaging of TNNT2-FUCCI Cardiomyocytes Shows Differences in Cell Cycle Progression With G2 Phase Arrest in Polyploidy

**Figure 2:**
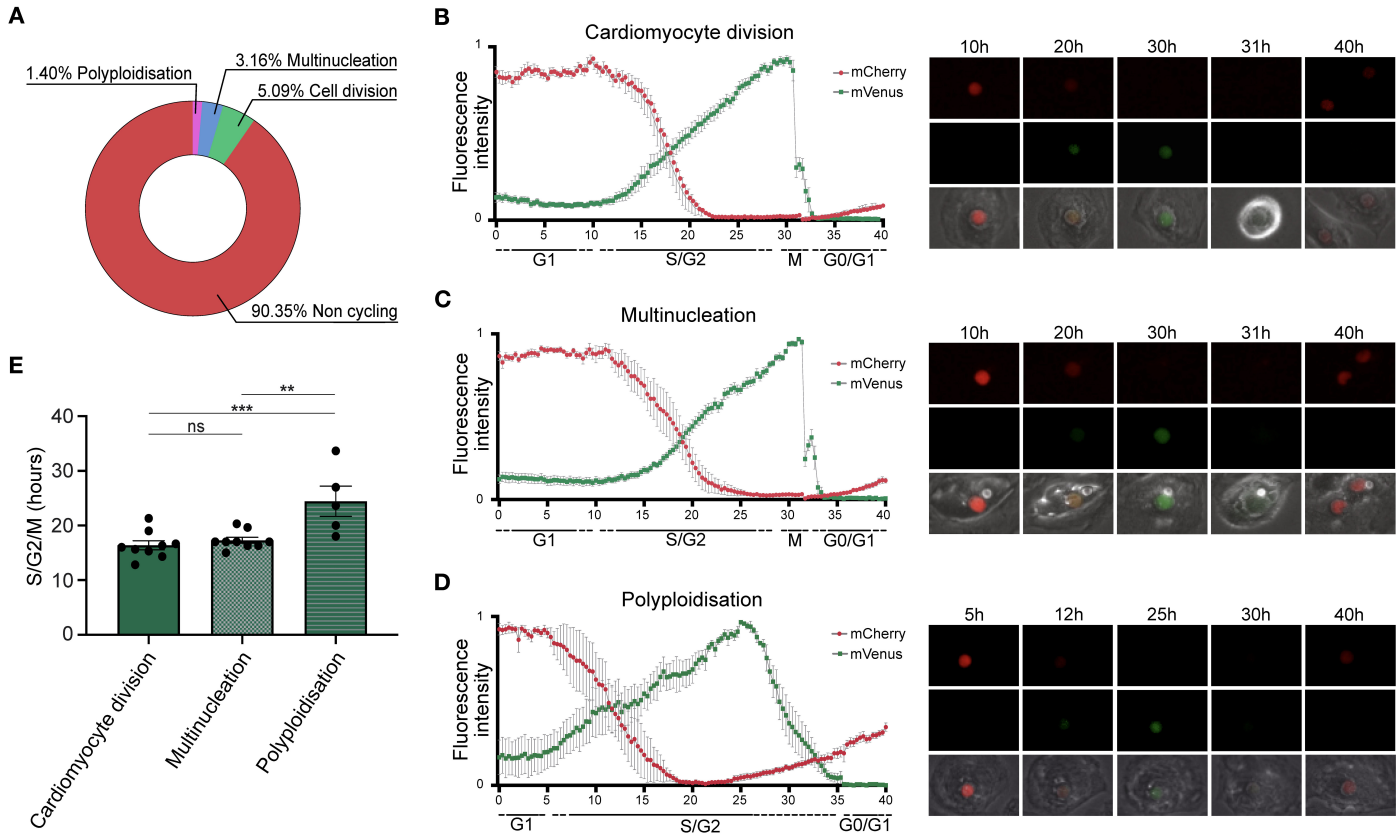
Quantification of cell cycle duration using live cell imaging of hiPSC-derived CMs. **(A)** Pie chart showing the percentage of non-cycling and cycling cardiomyocytes (*n* = 570) collected from 20 movies. **(B–D)** left panels show oscillation patterns of FUCCI fluorescence measured by live imaging in cardiomyocytes undergoing **(B)** cell division (*n* = 9), **(C)** multinucleation (*n* = 9), and **(D)** polyploidization (*n* = 5). Data show mean intensity ± SEM. Right panels show images of selected intervals of the movies. **(E)** Quantification of the total duration of the combined S, G2, and M phases shows a significant increase in cell cycle duration in cardiomyocytes undergoing polyploidization (*n* = 5) compared to multinucleation (*n* = 9) and polyploidization (*n* = 9). Data show mean intensity ± SEM. *P*-value was determined by one-way ANOVA. **p < 0.01, ***p < 0.001.

We tracked single TNNT2-FUCCI cardiomyocytes over 72 h (Figure 2) Among 570 cardiomyocytes analyzed, 90.4% ± 10.6% SEM did not show any cell cycle activity, 5.1 % ± 1.5% SEM underwent cytokinesis and cell division, 3.2% ± 1.5% SEM became binucleated, and 1.40% ± 0.8% SEM underwent polyploidization (Figure 2A). We could not detect any binucleated or polyploid cardiomyocyte undergoing cell division, supporting the notion that mainly diploid mononucleated cardiomyocytes show proliferative capacity (9). Next, we plotted the FUCCI oscillation pattern for cardiomyocytes undergoing cell division, multinucleation and polyploidization (Figures 2B–D and Supplementary Movies 1–3). In all three cycling populations, mVenus-geminin fluorescence started to increase concomitant with the reduction of the mCherry-Cdt1 signal, marking the end of the G1 phase and the beginning of the S/G2 phase. In early mitosis, mVenus fluorescence dropped sharply concomitant with nuclear envelope breakdown in dividing and multinucleating TNNT2-FUCCI cardiomyocytes (Figures 2B,C). The average duration of the S/G2/M phase was 16.38 h ± 0.84 SEM h in dividing cells and 17.29 h ± 0.55 SEM h in multinucleating cells, with no significant difference between the two populations (p > 0.05, Figure 2E). In contrast, polyploid cells showed a different FUCCI oscillation pattern. An increase in the mCherry signal was detected before the loss of mVenus, which dropped slowly, suggesting G2 phase arrest without activation of the anaphase-promoting complex (26). Accordingly, no mitotic features, such as cell rounding or cytoplasmic localization of mVenus, were detected (Figure 2D). Moreover, mVenus expression was detected over a significantly longer period in polyploid cells compared to dividing (*p* = 0.0009) and multinucleating (*p* = 0.0029) cells, with an average duration of the S/G2 phase of 24.5 h ± 2.77 h SEM (Figure 2E). These data suggest that hiPSC-derived CMs that become polyploid do not enter mitosis and remain arrested in theG2 phase.

### Live Image-Based TNNT2-FUCCI Screening Identifies Cell Cycle Activators

**Figure 3:**
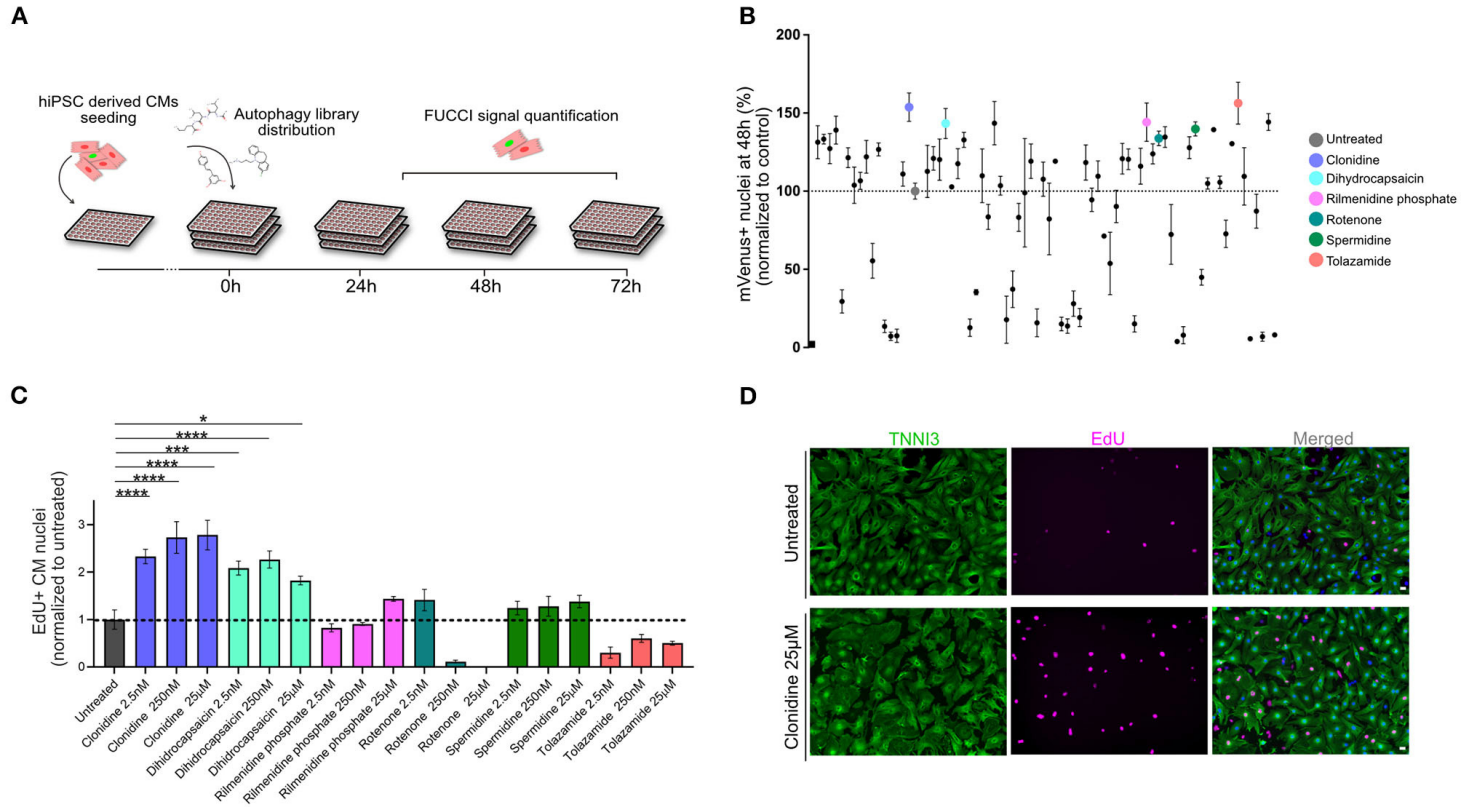
Live-image-based TNNT2-FUCCI hiPSC-derived cardiomyocyte screen identifies compounds that induce cell cycle activity. **(A)** Schematic drawing of the screening experiment. hiPSC-derived CMs were seeded in 96-well plates and stimulated with 94 compounds of an autophagy-related compound library at a concentration of 25 μM. FUCCI signal was documented at 24 h, 48 h, and 72 h. **(B)** The effect of each compound is shown as the percentage of mVenus^+^ nuclei relative to the control at the 48 h time point. Results are from one screen with three replicate wells on separate plates. Error bars represent STDEV between the triplicates. Six compounds that increased the percentage of mVenus^+^ cardiomyocyte nuclei were subjected to further validation, these are indicated with colors. **(C)** Six compounds were further tested in mNCMs at three different concentrations (2.5 nM, 250 nM and 25 μM). Cell cycle activity was validated by EdU incorporation and subsequent detection at 72 h. The experiment was repeated three times, each with three replicates wells. Values are mean ± SEM. P-values were determined by one-way ANOVA. *p < 0.05, ***p < 0.001, ****p < 0.0001. **(D)** Representative immunocytochemistry images of untreated and clonidine-treated mNCMs with EdU incorporation detected in magenta and cardiac troponin I in green. Scale bar, 20 μm.

Next, we devised a live screening platform in which we probed TNNT2-FUCCI cardiomyocytes for cell cycle entry using a library of 94 autophagy-related compounds (Figure 3A, and Methods). TNNT2-FUCCI live cardiomyocytes were imaged, and mVenus and mCherry nuclear fluorescence was documented at 24 h, 48 h, and 72 h (Figure 3A). Proproliferative activity was determined as the percentage of mVenus^+^ (S/G2/M phase) cardiomyocyte nuclei and normalized to the control group at all three time points (Figure 3B and Supplementary Figures 3A,B). The acquisition time point of 48 h with the smallest coefficient of variance (24 h, 48 h, 72 h; 13.1%, 12.1%; 28.0%, respectively) was chosen to select six pro-proliferative candidates. We selected the six pro-proliferative candidate compounds based on their increase in the percentage of mVenus^+^ nuclei and their biological significance (see Methods and Supplementary Figures 3A,B).

### Validation of Pro-proliferative Candidate Compounds in mNCMs

The six selected compounds were tested at three different concentrations to establish potential concentration dependencies (2.5 nM, 250 nM, 25 μM) in mNCMs. Cell cycle activation was validated by EdU incorporation after 72 h which can detect DNA synthesis during S-phase (Figure 3C). We found two compounds that significantly increased cell cycle activity at all tested concentrations. Of these, the alpha-adrenergic and imidazoline receptor agonist clonidine showed the most pronounced concentration-dependent effect (2.78-fold increase compared to untreated, *p* < 0.0001) on cell cycle activity in mNCMs (Figures 3C,D and Supplementary Figure 3C). Hence, we continued to explore the pro-proliferative potential of clonidine (Supplementary Figure 3D).

### Clonidine Triggers Proliferation in hiPSC-Derived CMs and Cell Cycle Activity in mNCMs

**Figure 4:**
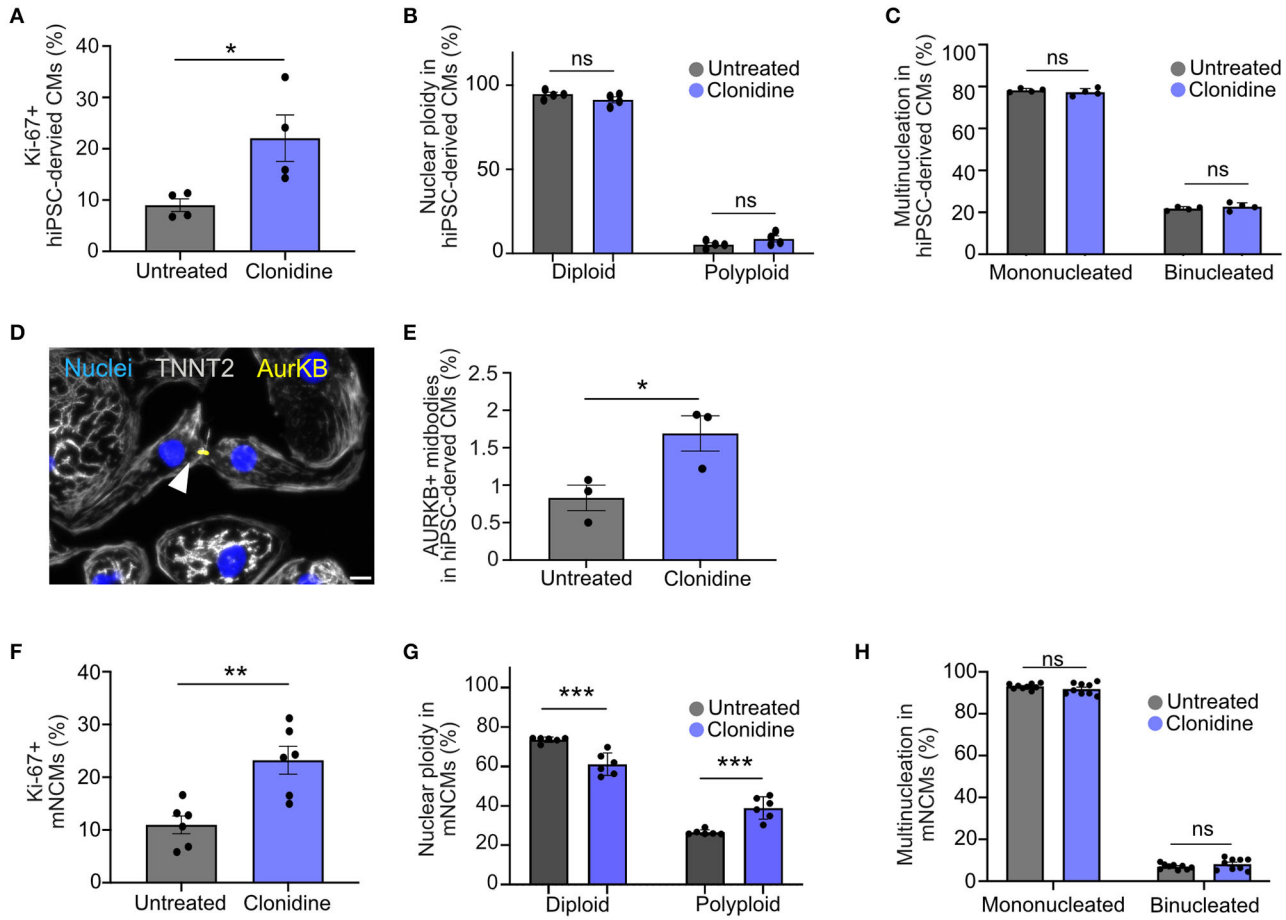
Effect of clonidine on cell cycle activity and proliferation in human and mouse cardiomyocytes **(A)** Quantification of Ki-67^+^ hiPSC-derived CMs after 72 h of clonidine treatment. *P*-values were determined by unpaired t-test. Datapoints represent four individual wells from one experiment. **(B,C)** Cell cycle activity induced by clonidine did not show any changes in **(B)** nuclear ploidy or **(C)** binucleation. Datapoints represent four individual wells with 11 fields of view analyzed per well. *P*-values were determined by two-way ANOVA. **(D,E)** AurKB^+^ midbodies, indicative of late phase cytokinesis, increased significantly in clonidine-treated hiPSC-derived CMs. Datapoint represent three individual wells. Scale bar, 20 μm. *P*-values were determined by unpaired *t-test*. **(F)** Clonidine significantly increased the percentage of Ki-67^+^ mNCMs. Datapoints represent six individual wells, P-values were determined by unpaired *t-test*. **(G)** Quantification showed an increase in nuclear ploidy in mNCMs treated with clonidine. Datapoints represent six individual wells. *P*-values were determined by two-way ANOVA. **(H)** Ratios of binucleated mNCMs were not altered by clonidine treatment. P-values were determined by two-way ANOVA. Values are mean ± SEM. *P < 0.05, **p < 0.01, ***p < 0.001. Datapoints represent nine wells with three fields of view analyzed for each well.

We assessed whether clonidine-induced cell cycle activity results in proliferation or polyploidy in hiPSC-derived CMs (Figures 4A–E). Cell cycle activity was first determined by Ki-67 expression. A cell cycle related gene, which expression is gradually increased during cell cycle progression, reaching a maximum at G2/S (27). Although there are reports that Ki-67 expression is linked to DNA damage response (27, 28), Ki-67 is widely used to demonstrate cell cycle activity (28). We could show a high fidelity of Ki-67 in detecting cell cycle progression (S/G2/M) in neonatal mice (14). We measured an increase in cell cycle activity after clonidine treatment through Ki-67 expression from 9.0% ± 1.23% SEM to 22.0% ± 4.51% SEM at 72 h after treatment in hiPSC-CMs (*p* = 0.03, Figure 4A). Clonidine did not cause any changes in polyploidy (*p* > 0.05, Figure 4B, and Supplementary Figure 4A) or in binucleation (*p* > 0.05, Figure 4C), suggesting that most clonidine-induced cell cycle activity results in proliferation. Consistent with these findings, we found an increase in aurora B kinase (AurKB)-positive midbodies, indicative of late phase cytokinesis, from 0.83% ± 0.17% SEM in untreated hiPSC-derived CMs to 1.69% ± 0.24% SEM in clonidine-treated hiPSC-derived CMs (*p* = 0.04, Figures 4D,E).

Next, we investigated whether clonidine elicits similar effects in mNCMs that are more restricted in their capacity to proliferate. We found an increase in cell cycle activity, measured through Ki-67 expression, from 10.97% ± 1.68% SEM in untreated mNCMs to 23.23% ± 2.63% SEM in clonidine-treated mNCMs (*p* = 0.002, Figure 4F). The ratio of binucleated cardiomyocytes was not altered by clonidine treatment (*p* > 0.05, Figure 4H), but we found an increase in nuclear ploidy with an increase in the tetraploid fraction from 26.52% ± 0.54% SEM in untreated mNCMs to 38.93% ± 2.32% SEM in clonidine-treated mNCMs (*p* = 0.0004, Figure 4G, Supplementary Figure 4B), suggesting that a substantial proportion of clonidine-induced cell cycle activity can be attributed to nuclear polyploidy. Additionally, we performed AurKB staining on clonidine-treated and control mNCMs. Although we observed AurKB^+^ cardiomyocyte nuclei in all cultures (Supplementary Figure 4C), we could not detect any positive midbodies in these cultures (more than 10,000 cardiomyocytes analyzed), suggesting that clonidine does not stimulate cytokinesis in cell cycle-active mNCMs. In agreement with this, cardiomyocyte cell count did not show any significant increase (*p* > 0.05, Supplementary Figure 5D) in the number of cardiomyocytes after clonidine treatment.

### Clonidine Induces Cell Cycle Activity in the Neonatal Mouse Heart

**Figure 5:**
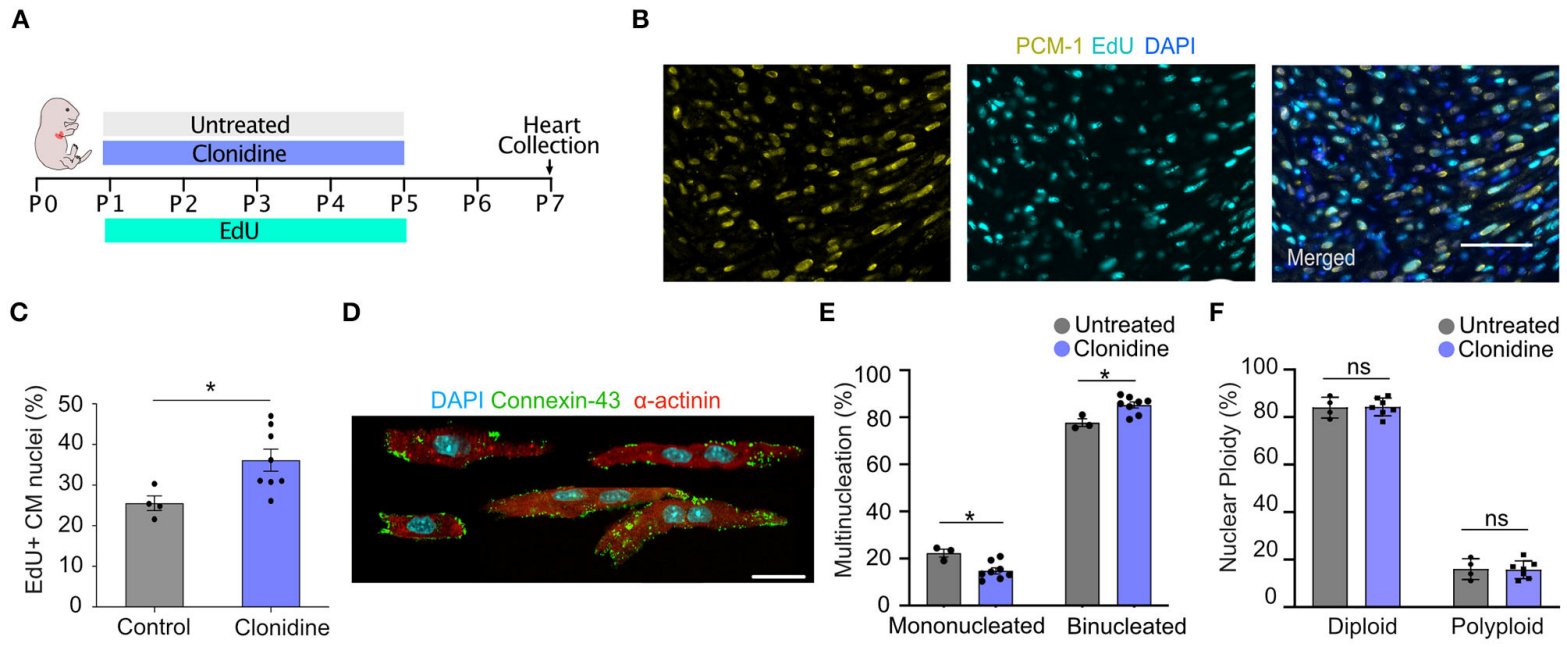
Clonidine induces cell cycle activity in neonatal mice. **(A)** Neonatal mice were subjected to four days (P1-P5) of clonidine (60 ng/pup) and EdU treatment (20mg/kg). Pups were sacrificed and hearts were collected at P7. **(B,C)** Quantification of EdU^+^ cardiomyocyte (PCM-1^+^) nuclei in sectioned mouse hearts showed an increase in EdU incorporation in clonidine-treated neonatal mice. Three sites per heart section were imaged and a minimum of 100 cardiomyocytes nuclei were analyzed per sample. Each point represents a biological replicate, clonidine n = 8, untreated n = 4. Values are mean ± SEM. P-value was determined by unpaired *t-test*. *P < 0.05. Scale bar, 50 μm. **(D)** Shows compilation of individual representative images of digested P7 cardiomyocytes stained with α-actinin and connexin-43. Scale bar, 20 μm. **(E)** Quantification of mono and binucleated cardiomyocytes with clonidine treatment (*n* = 8) and untreated (*n* = 3). At least 100 cardiomyocytes per biological replicate were analyzed. Values are mean ± SEM. *P*-value was determined by unpaired *t-test*. *P < 0.05. **(F)** Quantification of nuclear ploidy on cardiomyocytes with clonidine treatment (*n* = 8) and untreated (*n* = 4) shows no significant difference. At least 422 cardiomyocyte nuclei were analyzed per mouse.

To explore whether clonidine triggers cell cycle activity in the mouse heart, similar to what we found *in vitro*, we administered clonidine to neonatal mice. Clonidine was given along with EdU daily from P1 to P5 (60 ng/day), and the hearts were collected for analysis at P7 (Figure 5A). The number of EdU^+^ cardiomyocyte nuclei (EdU^+^/PCM-1^+^) was significantly increased in the clonidine-treated group (36.15% ± 2.72% SEM) compared to control animals (25.54% ± 1.80% SEM) (*p* = 0.02, Figures 5B,C), demonstrating that clonidine triggers cardiomyocyte cell cycle activity as more cells have entered S-Phase in both *in vitro* and *in vivo* mouse neonatal hearts. In contrast to neonatal cardiomyocyte *in vitro*, nuclear ploidy levels remained constant (Figures 5D,F and Supplementary Figure 5C), whereas the fraction of mononucleated cardiomyocytes was reduced in clonidine treated animals from 14.8% ± 1.3 SEM%, compared to 22.3% ± 1.7% SEM in controls (*p* = 0.01, Figure 5E), demonstrating mitotic activity without cytokinesis.

As alpha adrenergic signaling is not restricted to cardiomyocytes (29), we explored the possibility that cell cycle activity is altered in other cell types of the heart. Whereas, we did not see any changes in fraction of EdU^+^ endothelial cells (control: 29.4% ± 2.8% SEM vs. clonidine: 30.1% ± 1.7% SEM, *p* = 0.85), we found an increase in smooth muscle cell cycle activity from 44.2% (29.4–46.9%, interquartile range) in controls to 59.3% (51.9–61.7%, interquartile range) in clonidine treated animals (*p* = 0.04, Supplementary Figures 5D–F).

## Discussion

Heart failure is among the leading causes of death in the Western world, and currently available treatment is limited to salvaging existing cardiomyocytes or heart transplantation. Augmenting the proliferation of existing cardiomyocytes is often proposed as a promising future strategy for reverting disease progression (30). To achieve this goal, detailed knowledge of the human cardiomyocyte cell cycle and how it can be manipulated is crucial. Here, we generated TNNT2-FUCCI cardiomyocytes to reveal cell cycle kinetics in human cardiomyocytes undergoing proliferation, binucleation and polyploidization. To show the versatility of TNNT2-FUCCI, we devised a live cell screening platform to assess the pro-proliferative effects of compounds from an autophagy library. Using this platform, we identified clonidine as a compound that induces cell cycle activity in cardiomyocytes, resulting in the proliferation of hiPSC-CMs.

### Generation and Characterization of a hiPSC Line With the Cardiomyocyte-Specific TNNT2-FUCCI Reporter

The identification of cycling cardiomyocytes is critical for studying cardiomyocyte proliferation (10, 14, 31–34). Here, we generated the TNNT2-FUCCI hiPSC reporter line and differentiated it into cardiomyocytes. Consistent with previous data, we showed that inactivation of one TNNT2 locus by FUCCI knock-in did not cause structural impairments in cardiomyocytes (35). After 6 days in culture, we found the first cells expressing TNNT2-FUCCI along with the appearance of beating cardiomyocytes at Day 8 of differentiation (17). We found a complete overlap of cyclin-dependent kinase 1 (CDK1), which oscillates in the cell cycle and shows activity in S/G2/M phases (36), with mVenus-geminin^+^ cardiomyocytes, verifying the fidelity of geminin expression and thereby with the TNNT2-FUCCI system.

### Single-Cell Live Imaging of Cycling Cardiomyocytes

Video time-lapse microscopy is considered the gold standard to unequivocally determine the outcome of the cardiomyocyte cell cycle as division, multinucleation or polyploidization (10, 14, 37). Here, we combined time-lapse microscopy with our TNNT2-FUCCI sensor to determine the length and distinct phases of the cardiomyocyte cell cycle. We found that the S/G2/M phase took approximately 18 h for both dividing and binucleating cells with similar TNNT2-FUCCI fluorescent oscillation patterns, documenting a slightly longer cell cycle length than in mouse embryonic E14.5 cardiomyocytes (10.4 h) (15) and in mNCMs at P0 (15.1 h) (14). In contrast, human cardiomyocytes undergoing polyploidy showed a longer S/G2/M phase duration of ~25 h and prominent changes in the TNNT2-FUCCI fluorescence intensity pattern, exemplifying the difficulty in estimating annual cell cycle activity rates based on a fixed duration of cell cycle length (38, 39). Furthermore, our data suggest that polyploidy is not only a result of karyokinesis failure (40) because we did not observe nuclear envelope breakdown in cardiomyocytes but can also be a result of G2-phase arrested cycling cardiomyocytes.

### Live TNNT-FUCCI Screening Platform

Here, we developed a fluorescence-based live imaging screening platform based on the TNNT2-FUCCI system. This screening platform does not require immunofluorescence staining and eliminates the need for total cell counts and the incorporation of nucleotide analogs (e.g., BrdU and EdU). Live imaging of cycling cardiomyocytes allows for multiple imaging time points to establish the efficacy time course of pro-proliferative compounds. Due to the cardiomyocyte specificity of the TNNT2-FUCCI system, we eliminated false-positive detection of proliferative events originating from non-depleted cycling non-cardiomyocytes. Moreover, TNNT-FUCCI cardiomyocyte specificity allows sophisticated cocultures of cardiomyocytes with other cell types and could even be combined with recently developed cardiac organoids (41, 42). Our platform can be used with any type of library to identify pro-proliferative candidates. In this study, we subjected TNNT2-FUCCI hiPSC-derived CMs to a library of compounds regulating autophagy. Autophagy has been implicated in heart regeneration (43) and exerts numerous roles in myocardial stress responses (44). Autophagy reduces cellular stress caused by reactive oxygen species, which in turn plays a role in cardiomyocyte cell cycle exit (45).

### Validation of Screen-Identified Compounds in mNCMs

We selected six compounds for further validation in mNCMs (Supplementary Table 1), which are considered more mature than iPSC-derived CMs (18), with a partial loss of their capacity to proliferate. We found that only two of the six compounds significantly enhanced cell cycle activity, potentially due to higher levels of maturation in mNCMs compared to hiPSC-derived CMs and cross-species variation in cardiomyocyte physiology. One of these two compounds was dihydrocapsaicin, an analog of the active component of chili pepper with documented effects on cardiomyocytes, including autophagy induction (46) and attenuation of mitochondrial function (47).

The most robust and dose-dependent effect on cardiomyocyte cell cycle entry was elicited by the alpha-adrenergic and imidazoline agonist clonidine. Although clonidine is an alpha2-adrenergic agonist, it also shows binding to alpha1-adrenergic receptors (48). Furthermore, clonidine activates the imidazoline receptor, leading to a decrease in the level of cAMP in cells, resulting in downstream autophagy activation (49). Alpha-adrenergic receptors are predominantly found on smooth muscle cells of blood vessels, mediating vasoconstriction (29), but can also be detected on cardiomyocytes (50). Accordingly, we found an increase in cell cycle activity of smooth muscle cells in clonidine treated neonatal hearts. Single cell RNA sequencing data, re-analyzed from our previous study (14), showed expression of alpha1B and beta1 adrenergic receptors in P0 and P7 mNCMs. Apart from adrenergic receptor expression, we also found nischarin (Nisch), the mouse homolog of human imidazoline receptor I1 candidate (51), robustly expressed in mNCMs (Supplementary Figures 5A,B). We hypothesized that clonidine mainly elicits its pro-proliferative effects via direct alpha1 adrenergic receptor (50), and imidazoline receptor interaction on cardiomyocytes (52). Alpha1 adrenergic receptors are members of the G protein-coupled receptor superfamily. They activate mitogenic responses and regulate growth and proliferation of many cell types through several downstream signaling cascades such as the c-Jun NH2-terminal kinase (JNK) and the mitogen activated protein kinase (MAPK) pathways (53). Thus, we chose to further investigate whether clonidine treatment in addition to cell cycle entry also results in successful mitosis and cytokinesis.

Based on detected AurKB^+^ midbodies, clonidine promotes cytokinesis in hiPSC-derived CMs. While the increase in cell cycle activity did not alter the ratio between diploid and polyploid cells in hiPSC-derived CMs, in mNCMs, we did observe an increase in polyploid cardiomyocytes and no AurKB^+^ midbody formation after clonidine treatment. The latter indicates that clonidine-induced cell cycle activity was not productive and that for successful completion of mitosis and cytokinesis in mNCMs, additional stimuli are required. Cardiomyocyte maturation is linked to a number of processes including changes in structure, metabolism and gene expression, and plays a major role in cardiomyocyte cell cycle exit (54). Whereas, immature hiPSC-derived CMs still have the capacity to divide given optimal condition (55), mNCMs gradually lose their proliferative capacity during the first postnatal days (1, 2, 45). Silencing of cell cycle and cytokinesis related genes such as AURKB and ECT2 along with an activation of mitochondrial biogenesis and a metabolic switch to oxidative phosphorylation has been attributed to a proliferation-to-hypertrophy transition in the developing and neonatal heart (45, 56). These changes could explain why clonidine acts differently on hiPSC-derived CMs and mNCMs in this study.

In conclusion, we generated TNNT2-FUCCI hiPSCs and demonstrated that TNNT2-FUCCI hiPSC-derived CMs enable analysis of cell cycle entry and progression, providing a powerful platform for screening and validation of pro-proliferative candidates in human cardiomyocytes.

## Data Availability Statement

The original contributions presented in the study are included in the article/Supplementary Material, further inquiries can be directed to the corresponding author/s.

## Ethics Statement

The studies involving human participants were reviewed and approved by Ethikkommission an der Technischen Universität Dresden (BO-EK-38012020). Written informed consent for participation was not required for this study in accordance with the national legislation and the institutional requirements. The animal study was reviewed and approved by Stockholm Local Ethics Committee and Landesdirektion Sachsen.

## Author Contributions

FM, MB, WD, IS, and PT: methodology. FM, MB, WD, KN, SK, PT, and OB: investigation. FM, WD, and OB: conceptualization, writing—original draft, and writing—review and editing. FM, WD, KG, and OB: supervision. OB: funding acquisition. All authors contributed to the article and approved the submitted version.

## Funding

OB was supported by the Center for Regenerative Therapies Dresden, the Karolinska Institute, the Swedish Research Council, the Ragnar Söderberg Foundation, the Åke Wiberg Foundation, and the LeDucq Foundation.

## Conflict of Interest

The authors declare that the research was conducted in the absence of any commercial or financial relationships that could be construed as a potential conflict of interest.

## Publisher's Note

All claims expressed in this article are solely those of the authors and do not necessarily represent those of their affiliated organizations, or those of the publisher, the editors and the reviewers. Any product that may be evaluated in this article, or claim that may be made by its manufacturer, is not guaranteed or endorsed by the publisher.

## Supplementary Material

Supplementary Movie 1 Time-lapse recording of a TNNT2-FUCCI cardiomyocyte undergoing polyploidization.

Supplementary Movie 2 Time-lapse recording of a TNNT2-FUCCI cardiomyocyte undergoing cytokinesis.

Supplementary Movie 3 Time-lapse recording of a TNNT2-FUCCI cardiomyocyte undergoing binucleation.

Supplementary Figure 1 Characterization of TNNT2-FUCCI hiPSC line. **(A)** Schematic representation of the FUCCI technology. The FUCCI system is based on two cell cycle regulator proteins, Geminin and Cdt1, which are fused to a green (mVenus) and red (mCherry) fluorescent protein respectively. During the cell cycle, Geminin and Cdt1 are ubiquitinated by specific ubiquitin E3 ligases and are then targeted to the proteosome for degradation. During the G0/G1 phase, Geminin is degraded and only Cdt1 is present and therefore the nuclei of the cells appear red. During the G1/S transition and S phase both proteins are present and the nuclei appear yellow. During the G2/M phase Cdt1 is degraded and only Geminin tagged with mVenus is present, resulting in green nuclei. At the end of the M phase and at the beginning of G0/G1 phase both proteins are absent and the nuclei appear colorless. **(B)** The CRTD5-TNNT2-FUCCI hiPSC line was karyotyped by Giemsa banding and showed an intact chromosome set similar to the parental line. **(C)** Immunocytochemistry staining shows the capacity to differentiate into the three germ layers; markers are shown in green. **(D)** Flow cytometry analysis showing high levels of expression of the pluripotency markers OCT4, SOX2, SSEA4 and TRA1-60. Blue markers indicate no antibody control, and red indicates the antibody-stained sample. The percentages shown refer to antibody-stained sample.

Supplementary Figure 2 Characterization of TNNT2-FUCCI cardiomyocytes. **(A)** Imaging flow cytometry recordings of non-myocytes. All signals observed in the TNNT2^−^ fraction in the green and red fluorescence channels could be assigned to autofluorescence. **(B)** Co-staining of FUCCI cardiomyocytes with cyclin-dependent kinase-1 (CDK1) shows an overlap of mVenus^+^ and CDK1^+^ nuclei. Scale bar, 20 μm. **(C)** TNNT2-FUCCI cardiomyocytes showed similar sarcomere spacing patterns, as determined using TNNT2 and α-actinin staining. Datapoints represent the analysis from a single cardiomyocyte (*n* = 10), from a two well experiment. Scale bar, 20 μm. **(D)** Cell size was not compromised in FUCCI cardiomyocytes. Scale bar, 20 μm. Datapoints represent the analysis from a single cardiomyocyte (*n* = 30), from a two well experiment.

Supplementary Figure 3 Autophagy compound screen of TNNT2-FUCCI cardiomyocytes. **(A,B)** Plots showing additionally acquired timepoint of the screen. The effect of each compound is shown as the percentage of mVenus^+^ nuclei relative to the DMSO control (=100%). Timepoints of 24 h and 72 h were used to generate these graphs. Error bars represent STDEV between triplicates. **(C)** Quantification and representative immunocytochemistry images of untreated and clonidine (25 μM and 100 μM)-treated mNCMs with EdU incorporation in purple and cardiac troponin I in green. *N* = 6 individual wells. Scale bar, 50 μm. Values are mean ± SEM. ^*^*P* < 0.05, ^**^*p* < 0.01. **(D)** Schematic representation of the experimental strategy for the validation of Clonidine as an inducer of cell cycle activity in hiPSC-derived CMs and in mNCMs *in vitro* and *in vivo*.

Supplementary Figure 4 Ploidy of noncycling in mNCMs. **(A,B)** Histograms showing the DNA intensity distribution of non-cycling nuclei (cardiac troponin I^+^/Ki-67^−^) of **(A)** hiPSC-derived CMs and **(B)** mNCMs. Mean of 1203 ± -211 SEM cardiomyocyte nuclei were analyzed to generate these 4 histograms. **(C)** Nuclear AurKB staining (red) indicates G2/M activity of mNCMs (green, arrowheads). More than 10,000 cardiomyocytes were analyzed, but no AurKB^+^ midbody assembly at the site of abscission was detected. Arrowheads indicate nuclear localization of AurKB in cardiomyocytes. Scale bar 20 μm. **(D)** No significant difference in cardiomyocyte cell count was found after clonidine treatment. Datapoints represent individual wells (*n* = 9).

Supplementary Figure 5 Receptor expression and cell cycle activity in cardiomyocytes and non-cardiomyocytes *in vivo*. **(A,B)** Expression levels of adrenergic receptor and imidazolin-1 receptor genes measured by single cell RNA sequencing in ventricular cardiomyocytes from P0 **(A)** and P7 **(B)** neonatal mouse hearts (expression plotted as normalized log counts). Data taken from (14) **(C)** Histograms showing the DNA intensity distribution of P7 cardiomyocyte nuclei (troponin I^+^) untreated or with clonidine treatment. Mean of 19028 ± -13009 SEM cardiomyocyte nuclei were analyzed to generate the histograms. **(D)** Immunohistochemistry images showing smooth muscle cells (α-SMA) and endothelial cells (Isolectin B4) and cells that have incorporated EdU. Scale bar, 20 μm. **(E)** Quantification of percentage of EdU^+^ of α-SMA^+^ cells, Values are Median ± quartile. ^*^*P* = 0.04, Mann-Whitney U test shows a significant increase in cycling smooth muscle cells. **(F)** Quantification of percentage of EdU^+^ of isolectin B4^+^ cells, values are Mean ± SEM.

Supplementary Table 1 Characterization of candidate compounds.

Supplementary Graphical Abstract FUCCI-based live imaging platform reveals cell cycle dynamics and identifies pro-proliferative compounds in human iPSC-derived CMs.
