# Supplementary figures and images for: FUCCI-Based Live Imaging Platform Reveals Cell Cycle Dynamics and Identifies Pro-proliferative Compounds in Human iPSC-Derived Cardiomyocytes
Source: Front Cardiovasc Med. 2022 Apr 25;9:840147. doi: 10.3389/fcvm.2022.840147 (PMC9081338; doi:10.3389/fcvm.2022.840147)

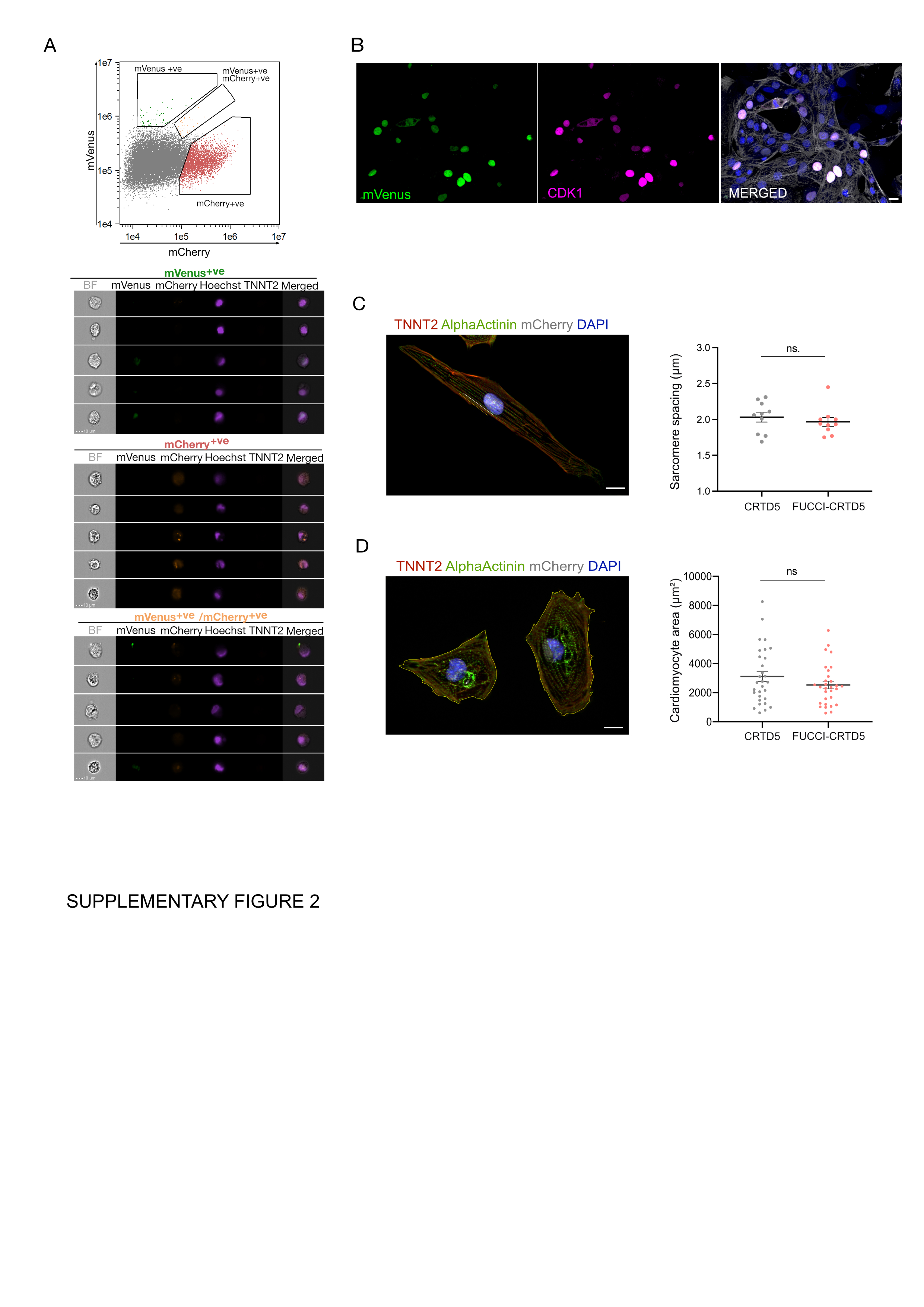

Supplement: Supplementary Figure 2 — Characterization of TNNT2-FUCCI cardiomyocytes. (A) Imaging flow cytometry recordings of non-myocytes. All signals observed in the TNNT2− fraction in the green and red fluorescence channels could be assigned to autofluorescence. (B) Co-staining of FUCCI cardiomyocytes with cyclin-dependent kinase-1 (CDK1) shows an overlap of mVenus+ and CDK1+ nuclei. Scale bar, 20 μm. (C) TNNT2-FUCCI cardiomyocytes showed similar sarcomere spacing patterns, as determined using TNNT2 and α-actinin staining. Datapoints represent the analysis from a single cardiomyocyte (n = 10), from a two well experiment. Scale bar, 20 μm. (D) Cell size was not compromised in FUCCI cardiomyocytes. Scale bar, 20 μm. Datapoints represent the analysis from a single cardiomyocyte (n = 30), from a two well experiment. [file Image_2.jpg]

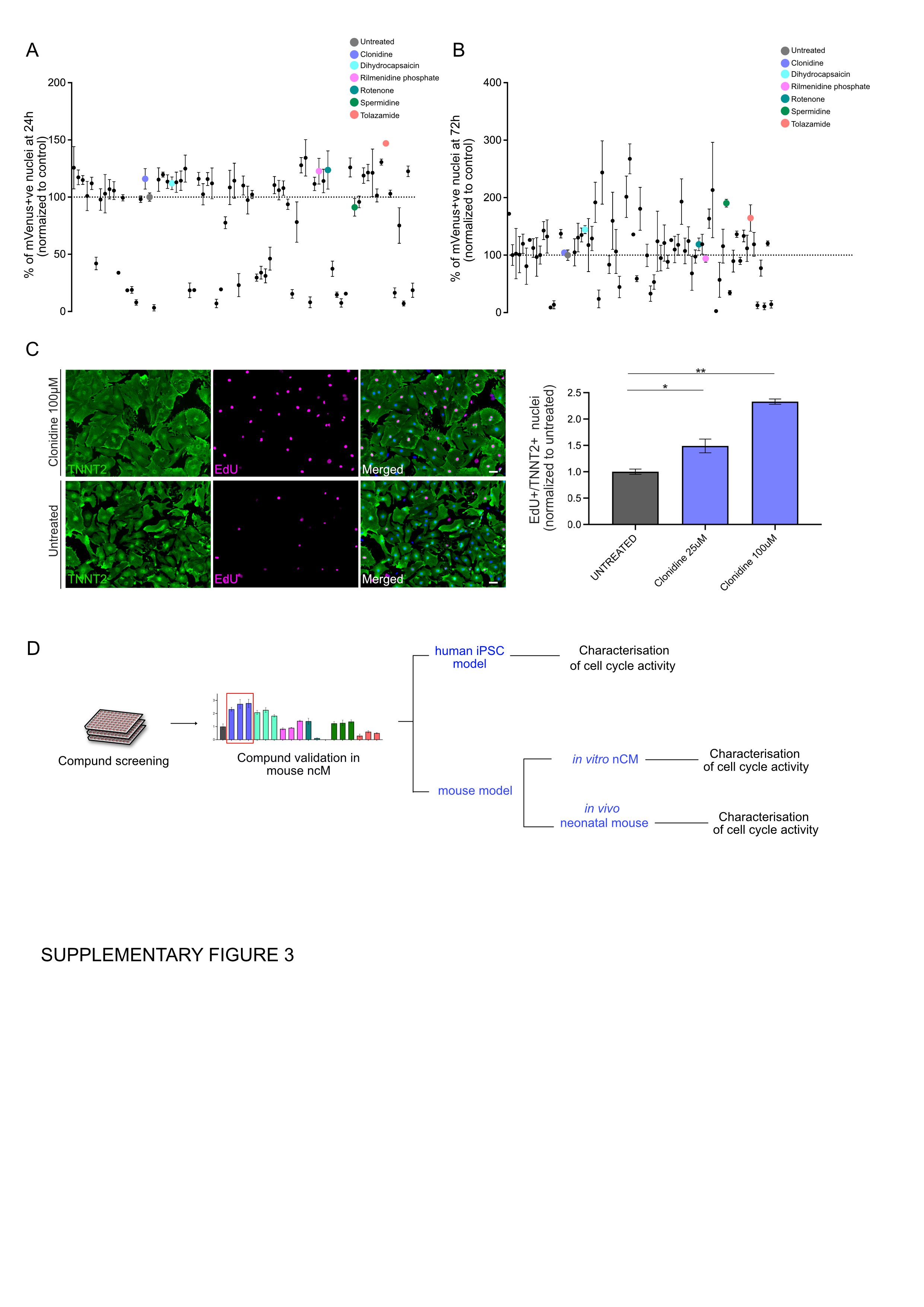

Supplement: Supplementary Figure 3 — Autophagy compound screen of TNNT2-FUCCI cardiomyocytes. (A,B) Plots showing additionally acquired timepoint of the screen. The effect of each compound is shown as the percentage of mVenus+ nuclei relative to the DMSO control (=100%). Timepoints of 24 h and 72 h were used to generate these graphs. Error bars represent STDEV between triplicates. (C) Quantification and representative immunocytochemistry images of untreated and clonidine (25 μM and 100 μM)-treated mNCMs with EdU incorporation in purple and cardiac troponin I in green. N = 6 individual wells. Scale bar, 50 μm. Values are mean ± SEM. *P < 0.05, **p < 0.01. (D) Schematic representation of the experimental strategy for the validation of Clonidine as an inducer of cell cycle activity in hiPSC-derived CMs and in mNCMs in vitro and in vivo. [file Image_3.jpg]

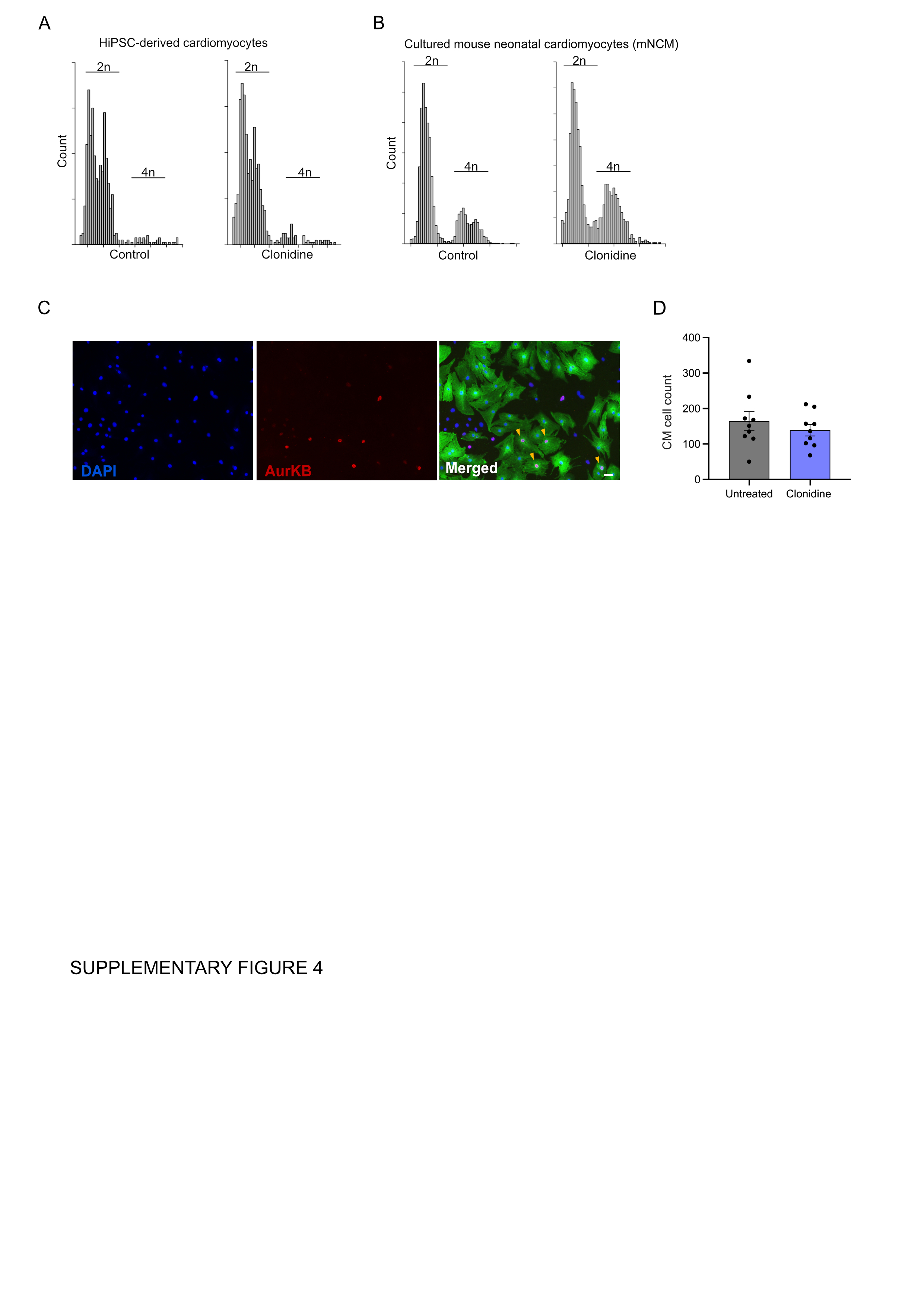

Supplement: Supplementary Figure 4 — Ploidy of noncycling in mNCMs. (A,B) Histograms showing the DNA intensity distribution of non-cycling nuclei (cardiac troponin I+/Ki-67−) of (A) hiPSC-derived CMs and (B) mNCMs. Mean of 1203 ± -211 SEM cardiomyocyte nuclei were analyzed to generate these 4 histograms. (C) Nuclear AurKB staining (red) indicates G2/M activity of mNCMs (green, arrowheads). More than 10,000 cardiomyocytes were analyzed, but no AurKB+ midbody assembly at the site of abscission was detected. Arrowheads indicate nuclear localization of AurKB in cardiomyocytes. Scale bar 20 μm. (D) No significant difference in cardiomyocyte cell count was found after clonidine treatment. Datapoints represent individual wells (n = 9). [file Image_4.jpg]

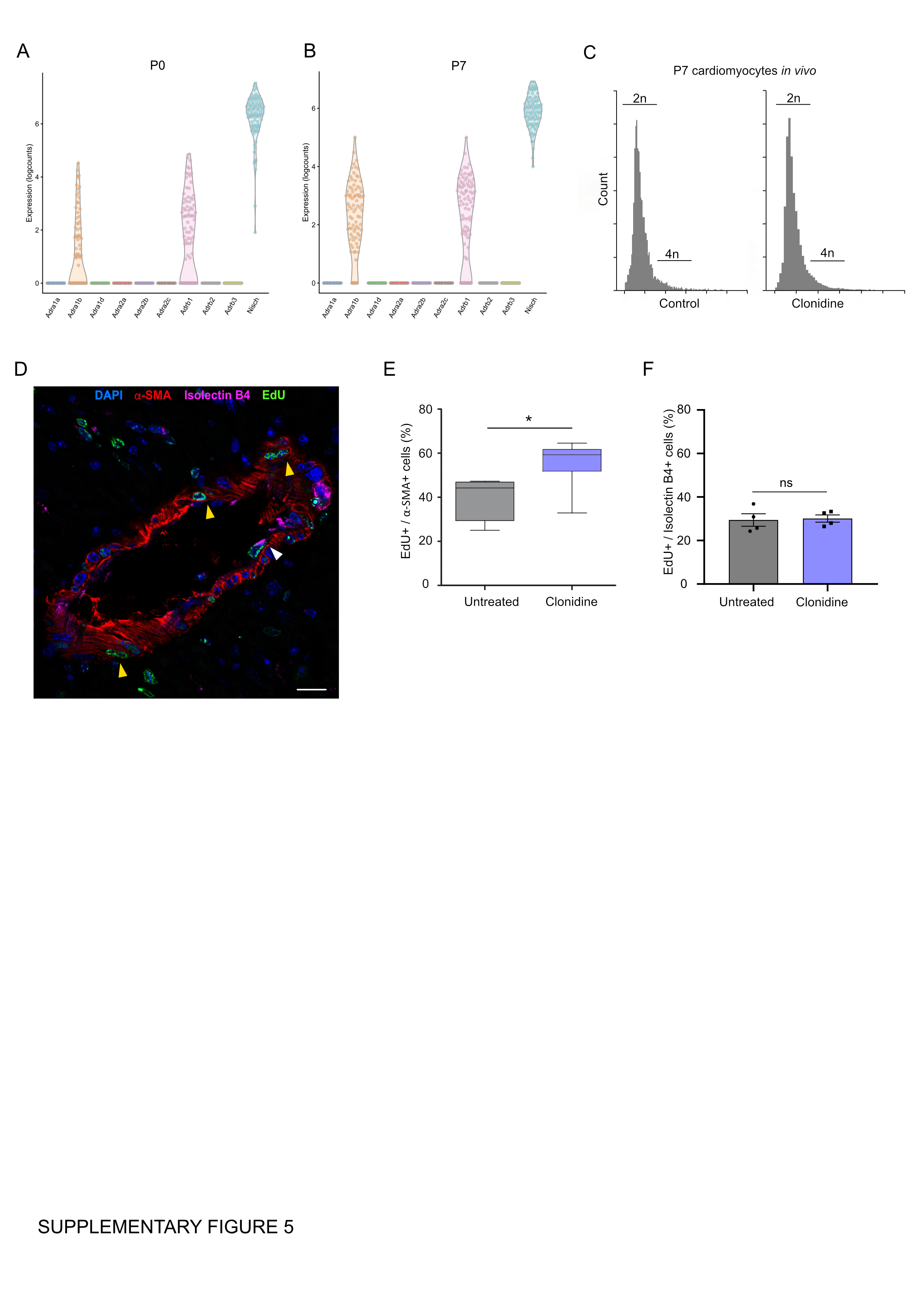

Supplement: Supplementary Figure 5 — Receptor expression and cell cycle activity in cardiomyocytes and non-cardiomyocytes in vivo. (A,B) Expression levels of adrenergic receptor and imidazolin-1 receptor genes measured by single cell RNA sequencing in ventricular cardiomyocytes from P0 (A) and P7 (B) neonatal mouse hearts (expression plotted as normalized log counts). Data taken from (14) (C) Histograms showing the DNA intensity distribution of P7 cardiomyocyte nuclei (troponin I+) untreated or with clonidine treatment. Mean of 19028 ± -13009 SEM cardiomyocyte nuclei were analyzed to generate the histograms. (D) Immunohistochemistry images showing smooth muscle cells (α-SMA) and endothelial cells (Isolectin B4) and cells that have incorporated EdU. Scale bar, 20 μm. (E) Quantification of percentage of EdU+ of α-SMA+ cells, Values are Median ± quartile. *P = 0.04, Mann-Whitney U test shows a significant increase in cycling smooth muscle cells. (F) Quantification of percentage of EdU+ of isolectin B4+ cells, values are Mean ± SEM. [file Image_5.jpg]
