# Supplementary material for: FUCCI-Based Live Imaging Platform Reveals Cell Cycle Dynamics and Identifies Pro-proliferative Compounds in Human iPSC-Derived Cardiomyocytes
Source: Front Cardiovasc Med. 2022 Apr 25;9:840147. doi: 10.3389/fcvm.2022.840147 (PMC9081338; doi:10.3389/fcvm.2022.840147)
Supplement: Supplementary Table 1 — Characterization of candidate compounds. [file Table_1.DOCX]

| **IUPAC Name** | **Name** | **Compound Purpose** | **Target/Mode of Action** |
| --- | --- | --- | --- |
| N-(2,6-dichlorophenyl)-4,5-dihydro-1H-imidazol-2-amine hydrochloride | Clonidine. hydrochloride | Autophagy inducer | I1R agonist, ↓cAMP |
| N-(dicyclopropylmethyl)-4,5-dihydro-1,3-oxazol-2-amine;phosphoric acid | Rilmenidine phosphate | Autophagy inducer | I1R agonist, ↓cAMP |
| (4-aminobutyl)(3-aminopropyl)amine | Spermidine | Autophagy inducer | ↓HATs |
| N-[(4-hydroxy-3-methoxyphenyl)methyl]-8-methylnonanamide | Dihydrocapsaicin | Autophagy inducer | ROS accumulation |
| 1-(azepan-1-yl)-3-(4-methylbenzenesulfonyl)urea | Tolazamide | Autophagy inhibitor | ATP-K+ channel antagonist |
| (1S,6R,13S)-16,17-dimethoxy-6-(prop-1-en-2-yl)-2,7,20-trioxapentacyclo[11.8.0.0³,¹¹.0⁴,⁸.0¹⁴,¹⁹]henicosa-3(11),4(8),9,14(19),15,17-hexaen-12-one | Rotenone | Autophagy inducer | mETC-complex1-inhib |

Compound information as presented in the SCREEN-WELL® Autophagy library by ENZO life sciences
