# Supplementary material for: FUCCI-Based Live Imaging Platform Reveals Cell Cycle Dynamics and Identifies Pro-proliferative Compounds in Human iPSC-Derived Cardiomyocytes
Source: Front Cardiovasc Med. 2022 Apr 25;9:840147. doi: 10.3389/fcvm.2022.840147 (PMC9081338; doi:10.3389/fcvm.2022.840147)
Supplement: Supplementary Graphical Abstract — FUCCI-based live imaging platform reveals cell cycle dynamics and identifies pro-proliferative compounds in human iPSC-derived CMs. [file Data_Sheet_1.PDF]

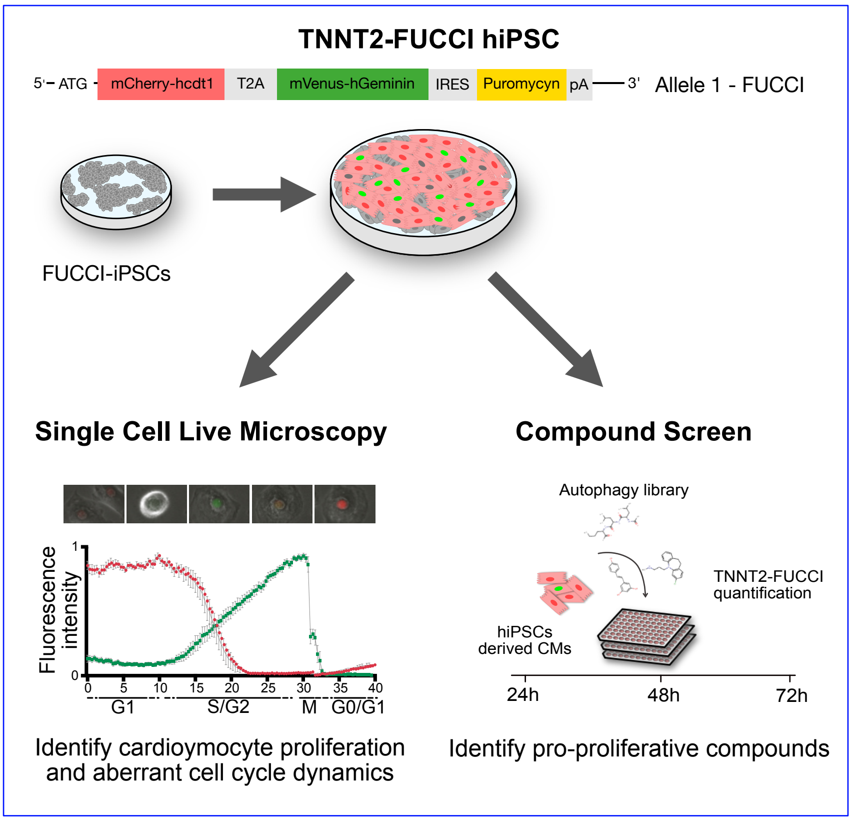
TNNT2-FUCCI hiPSC
5'– ATG
mCherry-hcdt1
T2A
mVenus-hGeminin
IRES
Puromycyn
pA
3'
Allele 1 - FUCCI
FUCCI-iPSCs
Single Cell Live Microscopy
Compound Screen
Fluorescence intensity
1
0
0
5
10
15
20
25
30
35
40
G1
S/G2
M
G0/G1
Autophagy library
TNNT2-FUCCI quantification
hiPSCs derived CMs
24h
48h
72h
Identify cardioymocyte proliferation and aberrant cell cycle dynamics
Identify pro-proliferative compounds
